# A multiplex platform for the identification of ovarian cancer biomarkers

**DOI:** 10.1186/s12014-017-9169-6

**Published:** 2017-10-10

**Authors:** Kristin L. M. Boylan, Kate Geschwind, Joseph S. Koopmeiners, Melissa A. Geller, Timothy K. Starr, Amy P. N. Skubitz

**Affiliations:** 1 Department of Laboratory Medicine and Pathology, School of Medicine, University of Minnesota, MMC 395, 420 Delaware Street, S.E, Minneapolis, MN 55455 USA; 2 Ovarian Cancer Early Detection Program, University of Minnesota, Minneapolis, MN USA; 3 Division of Biostatistics, School of Public Health, University of Minnesota, Minneapolis, MN USA; 4 Department of Obstetrics, Gynecology, and Women’s Health, University of Minnesota, Minneapolis, MN USA; 5 Masonic Cancer Center, University of Minnesota, Minneapolis, MN USA; 6 Department of Genetics, Cell Biology and Genetics, University of Minnesota, Minneapolis, MN USA

**Keywords:** Ovarian cancer, Biomarkers, Multiplex, CA125, HE4, Proseek^®^, Proximity extension assay

## Abstract

**Background:**

Currently, there are no FDA approved screening tools for detecting early stage ovarian cancer in the general population. Development of a biomarker-based assay for early detection would significantly improve the survival of ovarian cancer patients.

**Methods:**

We used a multiplex approach to identify protein biomarkers for detecting early stage ovarian cancer. This new technology (Proseek^®^ Multiplex Oncology Plates) can simultaneously measure the expression of 92 proteins in serum based on a proximity extension assay. We analyzed serum samples from 81 women representing healthy, benign pathology, early, and advanced stage serous ovarian cancer patients.

**Results:**

Principle component analysis and unsupervised hierarchical clustering separated patients into cancer versus non-cancer subgroups. Data from the Proseek^®^ plate for CA125 levels exhibited a strong correlation with current clinical assays for CA125 (correlation coefficient of 0.89, 95% CI 0.83, 0.93). CA125 and HE4 were present at very low levels in healthy controls and benign cases, while higher levels were found in early stage cases, with highest levels found in the advanced stage cases. Overall, significant trends were observed for 38 of the 92 proteins (p < 0.001), many of which are novel candidate serum biomarkers for ovarian cancer. The area under the ROC curve (AUC) for CA125 was 0.98 and the AUC for HE4 was 0.85 when comparing early stage ovarian cancer versus healthy controls. In total, 23 proteins had an estimated AUC of 0.7 or greater. Using a naïve Bayes classifier that combined 12 proteins, we improved the sensitivity corresponding to 95% specificity from 93 to 95% when compared to CA125 alone. Although small, a 2% increase would have a significant effect on the number of women correctly identified when screening a large population.

**Conclusions:**

These data demonstrate that the Proseek^®^ technology can replicate the results established by conventional clinical assays for known biomarkers, identify new candidate biomarkers, and improve the sensitivity and specificity of CA125 alone. Additional studies using a larger cohort of patients will allow for validation of these biomarkers and lead to the development of a screening tool for detecting early stage ovarian cancer in the general population.

**Electronic supplementary material:**

The online version of this article (doi:10.1186/s12014-017-9169-6) contains supplementary material, which is available to authorized users.

## Background

Ovarian cancer is the 5th leading cause of cancer deaths in women in the U.S. [1]. Early detection is the key to increased survival of patients, however, a screening tool that is adequately sensitive and specific for use in the general population has yet to be developed [2]. A need also exists for a reliable diagnostic method to distinguish between a benign mass and ovarian cancer [3].

For decades, researchers have been searching for protein biomarkers that can be incorporated into clinical tests to detect early stages of human cancers [4, 5]. In ovarian cancer, only a few biomarkers stand out, namely CA125 and HE4, which are currently approved by the FDA for monitoring recurrence of ovarian cancer [6–9]. These two biomarkers are not adequately sensitive or specific by themselves to screen the general population of women for ovarian cancer. A statistical method was developed to measure longitudinal changes in CA125 levels, and screening trials have recently been performed in which the risk of ovarian cancer algorithm has been used in combination with transvaginal ultrasound [10–12]. These trials showed an excellent specificity and positive predictive value in women with an average risk of ovarian cancer, and fared better than using a single cut-off for CA125 [13].

Over the decades, many groups have worked on the identification of ovarian cancer biomarkers. We and others have published hundreds of potential biomarkers identified by genomic and proteomic techniques, validated the findings, developed ELISAs, and screened sera…one biomarker at a time [5, 14–20]. Simultaneously screening dozens of biomarkers in a multiplex assay might improve the sensitivity and specificity to the extent that the assay could reasonably be used to screen an asymptomatic general population for early stage cases. For example, Trabert et al. [21] analyzed serum samples from women who later developed ovarian cancer on the Prostate, Lung, Colorectal, and Ovarian Cancer (PLCO) Screening Trial. They examined the levels of 60 immune and inflammation markers using Luminex bead-based commercial assay panels and found four proteins (C-reactive protein, interleukin-1α, interleukin-8, and tumor necrosis factor-α) were associated with an increased risk of subsequently developing ovarian cancer. Studies such as these suggest that it may be possible to identify a subset of biomarkers that detect the early stages of ovarian cancer at an acceptable rate for population screening.

In this study, we used technology developed by OLink Bioscience (Uppsala, Sweden), in which 92 cancer-related protein biomarkers are simultaneously quantified based on the proximity extension assay (PEA) [22–24]. This innovative technology combines the sensitivity of the polymerase chain reaction (PCR) with the specificity of antibody-based detection methods, allowing multiplex biomarker detection and high throughput quantification with similar assay precision to other multiplex detection methods [22–24]. The Proseek^®^ Multiplex Oncology Iv2 panel of cancer-related proteins encompasses 92 proteins that have been shown to be elevated in a variety of cancers. In particular, the panel contains CA125 and HE4, as well as 90 other cancer-related protein markers, including several that have been linked to ovarian cancer, such as ERBB2 [25, 26], ERBB3 [27], ERBB4 [28], vascular endothelial growth factor receptor (VEGFR) 2 [29], midkine [30–33], kallikrein 6 [34], kallikrein 11 [35], folate receptor-alpha [36], interleukin-6 [37–40], and transforming growth factor-alpha [41]. Proseek^®^ Multiplex Oncology I plates were recently used to identify a panel of biomarkers for the detection of early stage colorectal cancers [42, 43], while other studies have used Proseek^®^ plates for the discovery of bladder cancer biomarkers [44].

In this study, we report the first use of the Proseek^®^ Multiplex technology to determine its feasibility as a means to identify candidate biomarkers for early stage serous ovarian cancer. By using a multiplex approach in which 92 oncology-related proteins can be tested simultaneously, we set out to identify biomarkers that can be used in combination with CA125 and HE4 to develop a highly sensitive and specific assay for the detection of early stage ovarian cancer. This study focuses on high grade serous ovarian cancer, since it is the most prevalent and deadly subtype of ovarian cancer [45].

## Methods

### Serum samples

Blood samples were obtained by the University of Minnesota Tissue Procurement Facility staff with approval by the University of Minnesota Institutional Review Board under Protocol 0407M62504. After signing the consent form, blood was collected immediately before surgery from women with an abdominal mass suspected to be ovarian cancer (for the cases of benign ovarian disease, early stage ovarian cancer, and late stage ovarian cancer) or from women with benign non-gynecological health conditions (to serve as “healthy controls”) (e.g. eye surgery, hernia repair, hip replacement, and gall bladder removal). Samples were processed by standard operating procedures [14], divided into aliquots, and stored at − 80 °C. Serum samples were selected from each of four groups of patients: (1) 21 healthy controls, (2) 18 benign ovarian disease, (3) 21 early stage I/II serous ovarian cancer, and (4) 21 late stage III/IV serous ovarian cancer. Clinical, pathological and demographic information on subjects are presented in Table 1.

**Table 1:** Patient demographic and clinical information

	Diagnosis			
	Healthy	Benign ovarian disease	Early stage serous ovarian cancer	Late stage serous ovarian cancer
Number of cases	21	18	21	21
Age				
Mean	60.2 (7.3)	52.3 (14.9)	63 (11.5)	62.7 (8.9)
Median	59	48.5	64	64
Range	50–78	36–96	42–83	39–75
CA125 value				
Mean	2.5 (2.2)	47.5 (99.7)	1983.3 (5891.2)	1130 (1149.5)
Median	1.98	13	81.5	646
Range	0.19–10.17	4–413	17–22,780	124–3957
Race/ethnicity				
Caucasian	21	17	18	20
African American			2	
American Indian		1	1	
Pacific Islander				1
Stage				
I			11	
II			10	
III				18
IV				3
Grade				
1			1	1
2			3	5
3			16	14
Not specified			1	1

Mean values are shown with standard deviations in parentheses

Pre-operative CA125 values for the benign and cancer patients were obtained from medical records, having been performed in a clinical laboratory using CLIA certified assays, while the CA125 values for the healthy controls were generated using a commercially available kit (Abcam, Cambridge, MA; catalog #ab108653).

### Proseek^®^ technology and assay format

The reagents used in the Olink Proseek^®^ Multiplex 96-well plates are based on proximity extension assay technology [22, 42, 46], in which 96 oligonucleotide-labeled antibody pairs bind to their respective protein targets in the sample. When the oligo-tagged antibodies bind to the target protein in proximity to one another, a PCR reporter sequence is formed by DNA polymerization and subsequently detected and quantified using real-time PCR. This combination of antibody detection followed by PCR quantification permits the specific and sensitive analysis of 9216 proteins in one 96-well plate (96 proteins/well are measured; 92 biomarker proteins and 4 internal controls). The precision, reproducibility and scalability of the PEA assay have been previously described [22].

### Sample processing

We randomized the serum samples on a 96-well plate. As controls, we used a “Pooled Reference” containing equal volumes of serum from each of the samples. The Proseek^®^ plates also have 3 “Interplate controls” for data normalization between plates and 3 “Negative controls” to establish background levels. Ten microliters of sera were aliquoted into wells of a 96-well nonadherent plate (BioRad). One microliter of sera was subsequently transferred into a Proseek^®^ Multiplex Oncology Iv2 plate, and using Proseek^®^ reagents, the samples were processed in combination with the Fluidigm^®^ BioMark™ HD high-throughput PCR instrument [22]. Data generated from the Proseek^®^ Multiplex 96-well plate were analyzed, including normalization and linearization, per manufacturer protocol. The Proseek^®^ assay reports relative quantification on a log2 scale, as Normalized Protein eXpression (NPX) values, which are cycle threshold (C_t_) values normalized by the subtraction of values for the extension control. All assay characteristics including detection limits and measurements of assay performance and validation are available from the manufacturer’s website (http://www.olink.com/products/).

### Data analysis and statistics

Linear protein values were log-transformed and mean-centered to produce a data matrix of 81 patients by 92 proteins. Unsupervised clustering methods were applied to the data to identify clusters of proteins and visually evaluate their association with disease status. Unsupervised hierarchical clustering (uncentered correlation using centroid linkage) and K-means clustering (Euclidian distance, 5000 runs) were completed using Cluster 3.0 [47] and visualized using Treeview (v1.1.6r4) [48]. Principle component analysis was performed using the prcomp function in R.

The CA125 values obtained from the Proseek^®^ plate were compared to the ELISA values using Pearson’s correlation coefficient. Linear regression was performed to identify proteins that were differentially expressed between healthy, benign, early stage ovarian cancer, and late stage ovarian cancer patients. Trends for protein values were evaluated using linear regression with a conservative p < 0.001 as the cut-off for significance. The classification accuracy was evaluated using the receiver operating characteristic (ROC) curve and was summarized by the area under the ROC curve (AUC) and the sensitivity corresponding to a specificity of 0.95 (i.e. ROC(0.05)). The ROC curve and its summaries were estimated following the parametric binormal assumption [49]. We specifically focused on ROC(0.05) in order to compare the performance of our markers to existing multi-biomarker platforms for ovarian cancer. Confidence intervals for AUC and ROC(0.05) were calculated using the non-parametric bootstrap [50].

A multi-biomarker classifier for discriminating between sera from women with early stage ovarian cancer and healthy women was developed using supervised machine learning techniques. Variable selection was completed using the LASSO. Proteins were sequentially added to the model by manipulating the LASSO penalty parameter [51]. A naïve Bayes classifier was fit for each combination of proteins to allow for more flexible relationships between the proteins and cancer status in our predictive model [52]. The classification accuracy of our predictive model was summarized by the ROC curve and its summaries (AUC, ROC(0.05)). Leave-one-out cross validation was used to correct for the bias that results from validating our model on the same data that was used to build the model [52].

## Results

### Clustering based on multiplexed protein expression analysis

**Fig. 1:**
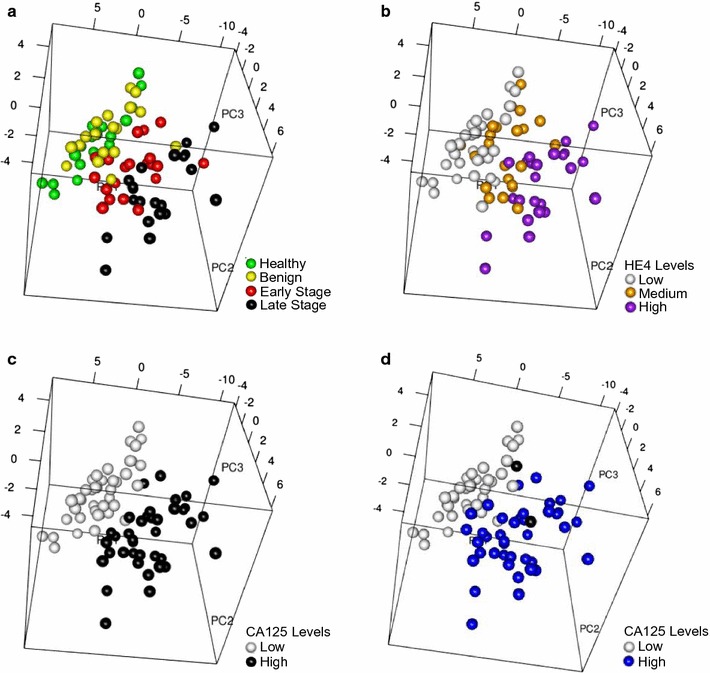
Principal component analysis of Proseek^®^ protein expression data. Principal component analysis plots were based on expression levels of 92 proteins measured in the sera of 81 patients using the Proseek^®^ Oncology I v2 Multiplex plates. **a** Colored circles represent healthy (green), benign (yellow), early stage ovarian cancer (red), and late stage ovarian cancer (black) serum samples. **b** Colored circles represent low (white), medium (orange) or high (purple) levels of HE4 in the serum samples based on Proseek^®^ data. **c** Colored circles represent low (white) or high (black) CA125 levels measured in the serum samples on the Proseek^®^ plates. **d** Colored circles represent CA125 levels measured in the serum samples by ELISA using the clinical cutoff values of < 35 U/ml (white) and > 35 U/ml (blue). The two black circles represent one benign patient and one early stage ovarian cancer patient who did not have ELISA CA125 values reported in their medical records

Using the mean-centered, log-transformed expression levels of all 92 proteins, we performed principal component analysis (PCA) to visually assess similarities and differences between samples and determine whether samples can be grouped. By coloring samples based on disease stage (healthy, benign, early, late) it is apparent that the differences in protein levels allow for segregation of the healthy/benign patients (green/yellow in Fig. 1a) from the early/late stage serous ovarian cancer patients (red/black in Fig. 1a). Not surprisingly, the two well-known biomarkers for advanced stage disease, CA125 and HE4, also segregate with the early/late stage ovarian cancer patients. In general, the healthy/benign patients had low Proseek^®^ HE4 values (Fig. 1b, white), while many of the early stage ovarian cancer patients had medium Proseek^®^ HE4 values (Fig. 1b, orange), and the late stage patients had high Proseek^®^ HE4 values (Fig. 1b, purple). Similarly, the healthy/benign patients had low Proseek^®^ CA125 values (Fig. 1c, white) while the early/late stage ovarian cancer patients had high Proseek^®^ CA125 values (Fig. 1c, black). This trend was also evident when examining the distribution of CA125 values as determined by ELISA (Fig. 1d); the healthy/benign patients tended to have ELISA CA125 values less than the clinical cutoff value of 35 U/ml, indicative of a “normal” CA125 value (Fig. 1d, white), whereas patients with early/late stage ovarian cancer had ELISA CA125 values greater than 35 U/ml, indicative of a risk for ovarian cancer (Fig. 1d, blue). These results illustrate the potential for using these proteins to discriminate by cancer status, and suggest that the first two principal components are mostly driven by CA125 and HE4.

**Fig. 2:**
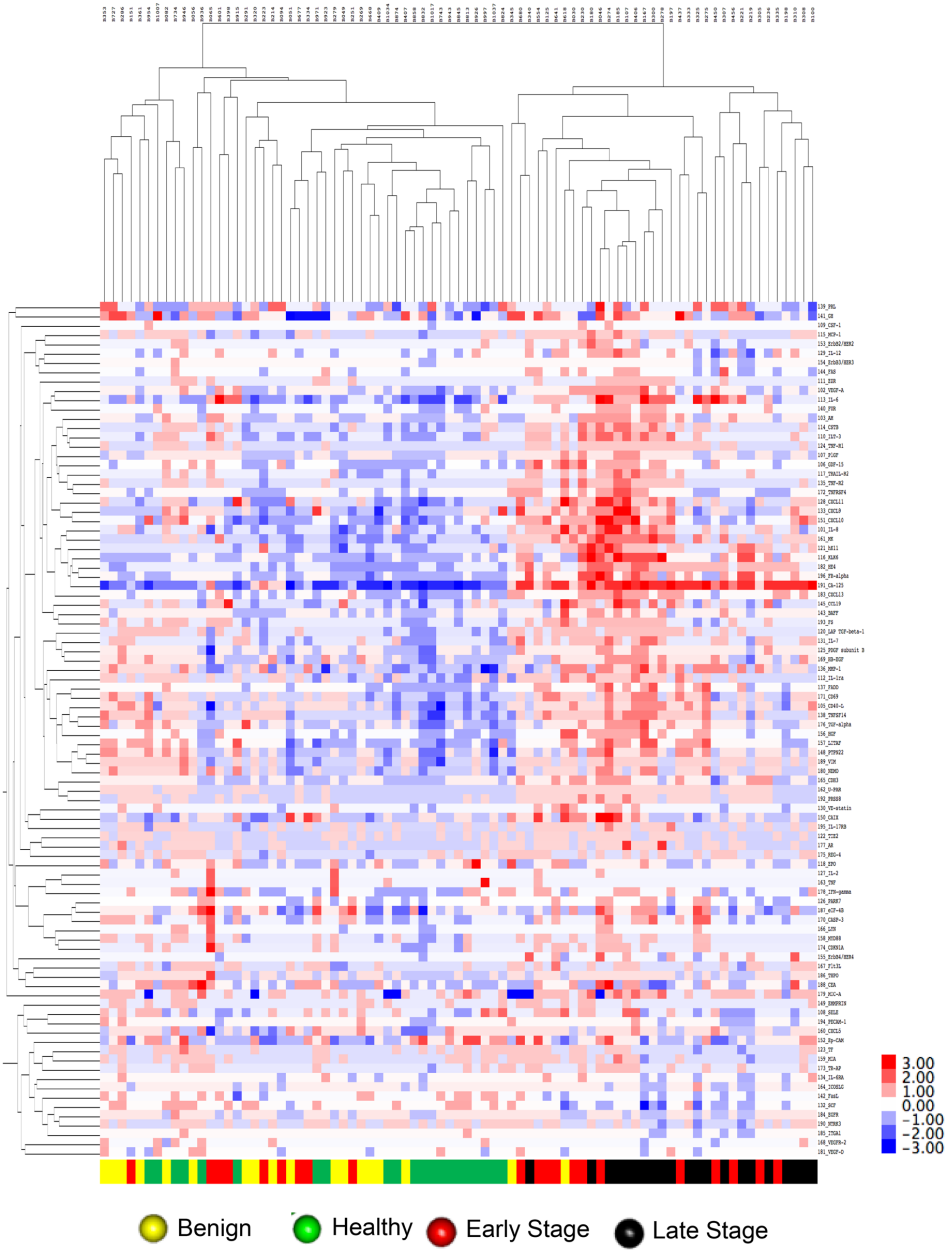
Unsupervised hierarchical clustering of Proseek^®^ protein expression data. Unsupervised hierarchical clustering was based on mean-centered log2 transformed protein expression data of 92 Proseek^®^ Oncology Iv2 proteins (Correlation uncentered, average linkage) measured in the sera of 81 patients. Dark red indicates high levels of the protein relative to the average value, white indicates the average value, and dark blue indicates that the protein levels are below average (shown in the color bar on the right hand side). Color bar at the bottom indicates patient classification: healthy (green), benign (yellow), early stage ovarian cancer (red), and late stage ovarian cancer (black)

Results of unsupervised hierarchical clustering can be found in Fig. 2. Similar to PCA analysis, all of the late stage, and the majority of early stage ovarian cancer patients clustered together (right cluster, Fig. 2), while 38 of the 39 healthy/benign samples clustered together (left cluster, Fig. 2). Visual analysis of the heat map indicates that over 20 proteins were elevated in the sera of women with early and late stage ovarian cancer, as depicted by red squares in the upper right quadrant of Fig. 2. Importantly, CA125 and HE4 are present in this quadrant, as well as other proteins that have been reported to be elevated in the sera of women with ovarian cancer, such as interleukin-6 [37–40], midkine [30–33], folate receptor-alpha [36], KLK6 [34], and hK11 [35]. In addition, many proteins that have not been reported in the literature as being associated with ovarian cancer were also present in this quadrant. Based on PCA and unsupervised hierarchical clustering, we show that multiplexed protein expression levels measured by the Proseek^®^ Multiplex Oncology Iv2 plate are a promising technology for distinguishing between healthy/benign and early/late stage ovarian cancer patients, and for the identification of candidate biomarkers.

### Correlations with ELISA values

**Fig. 3:**
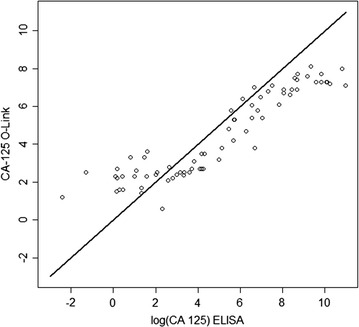
Comparison of the CA125 values obtained by ELISA versus the Proseek^®^ plate. Scatterplot comparison of the CA125 values for each of the 81 serum samples obtained by ELISA versus the Olink Proseek^®^ Oncology I v2 plate. Correlation coefficient of 0.89 (95% CI 0.83, 0.93)

We next compared the CA125 values obtained from the Proseek^®^ plate to the ELISA values using Pearson’s correlation coefficient. CA125 values measured by ELISA were analyzed on the log2 scale for consistency with the data obtained from the Proseek^®^ plate. The data from the Proseek^®^ plate exhibited a strong correlation with the ELISA data, with a correlation coefficient of 0.89 (95% CI 0.83, 0.93) (Fig. 3).

### Trends of protein values correlate with progression of ovarian cancer

**Fig. 4:**
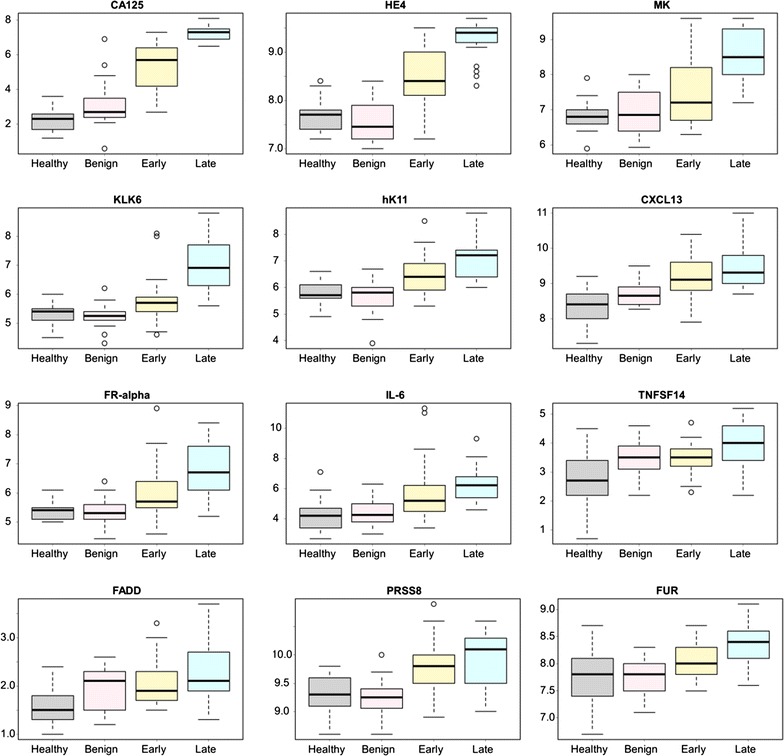
Proteins that showed an increasing trend in Proseek^®^ values in the four patient groups. The logarithmic output of the top 12 biomarkers that showed an increasing trend in values from healthy, to benign, to early stage serous ovarian cancer, to late stage serous ovarian cancer were graphed as quantile plots: CA125, HE4, MK, KLK6, hK11, CXCL13, FR-alpha, IL-6, TNFSF14, FADD, PRSS8, and FUR. Outliers are defined as any value higher or lower than 1.5 multiplied by the interquartile range. A complete ranking of all 92 proteins is shown in Additional file 1

Linear regression was performed to identify proteins that showed a trend of being differentially expressed between healthy, benign, early stage ovarian cancer, and late stage ovarian cancer patients. Overall, significant (p < 0.001) trends were observed for 38 of the 92 proteins, many of which have yet to be documented as candidate serum biomarkers for ovarian cancer (Additional file 1). The 12 most significant proteins were graphed as box plots (Fig. 4). As anticipated, CA125 and HE4 were present at very low levels in healthy controls and the benign cases, while higher levels were found in the early stage I/II cases, and the highest levels were found in the late stage III/IV cases. These data demonstrate that the Proseek^®^ technology can replicate the results previously established by conventional ELISA for these two known biomarkers. Other proteins that showed a significant increase in serum levels from healthy to late stage ovarian cancer included: midkine (MK), kallikrein 6 (KLK6), kallikrein 11 (hK11), CXC motif chemokine 13 (CXCL13), folate receptor-alpha (FR-alpha), interleukin-6 (IL-6), tumor necrosis factor superfamily member 14 (TNFSF14), FAS-associated death domain protein (FADD), prostasin (PRSS8), and furin (FUR). Three of these proteins, CXCL13, FADD, and TNFSF14 have not been reported in the literature as having an association with ovarian cancer, and may serve as novel candidate biomarkers for ovarian cancer. An additional 26 proteins showed significant differences (p < 0.001) across the four groups (Additional file 1). In some cases, the protein levels decreased in late stage ovarian cancer relative to the healthy controls (e.g. NT-3 growth factor receptor, integrin alpha-1, stem cell factor, epidermal growth factor receptor (EGFR), and interleukin-8).

### ROC/AUC for individual proteins for late stage ovarian cancer

**Fig. 5:**
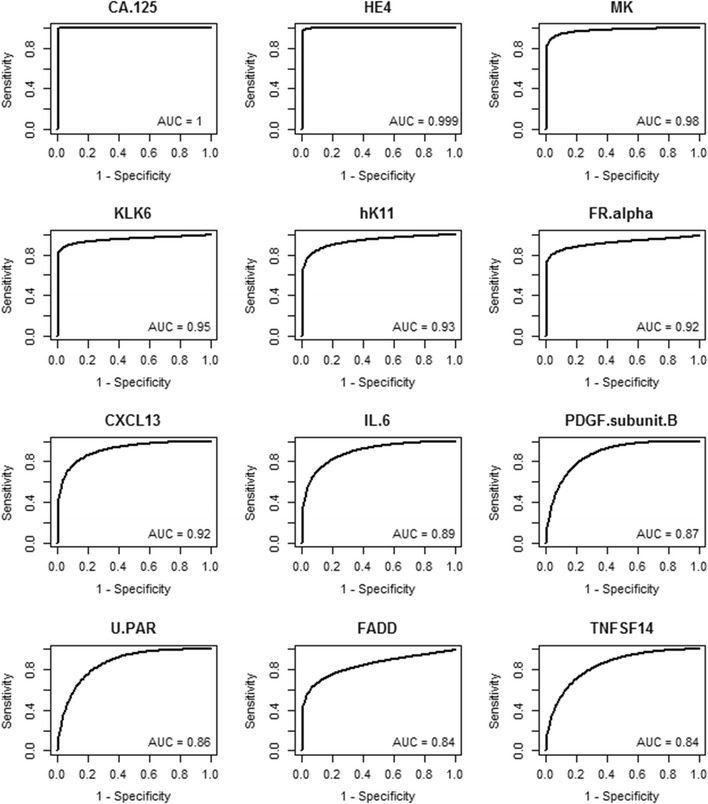
ROC curves for discriminating late stage ovarian cancer versus healthy women. ROC curves were graphed for the 12 proteins with the highest AUC values for discriminating late stage serous ovarian cancer versus healthy women. Data for the 25 proteins with the highest AUC values are shown in Table 2. Data for all 92 proteins is shown in Additional file 2

Receiver operating characteristic (ROC) curve analyses were completed for each of the 92 proteins to determine which proteins could discriminate between sera from women with late stage ovarian cancer and healthy women. The classification accuracy for each protein was summarized by the area under the ROC curve (AUC); ROC curves of the 12 proteins with the highest AUC values are graphed in Fig. 5. The 25 proteins with the highest AUC values are listed in Table 2, while the AUC values for all 92 proteins are listed in Additional file 2. The AUC for CA125 was 1.0 (95% CI 1.0, 1.0) and the AUC for HE4 was 1.0 (95% CI 0.99, 1.00) (Fig. 5). In total, 51 proteins had an estimated AUC of at least 0.70 and were significantly associated with cancer status. Many of the same proteins that were identified by unsupervised hierarchical clustering (Fig. 2) and by linear regression analysis (Fig. 4; Additional file 1), were also significantly higher in the late stage ovarian cancer patients relative to the healthy women, e.g. MK, KLK6, hk11, CXCL13, FR-alpha, IL-6, and FADD. The sensitivity at 95% specificity was also calculated and is shown for the top 25 proteins in Table 2, and for all 92 proteins in Additional file 2. CA125 had the highest sensitivity with a value of 1.0 (95% CI 1.0, 1.0), while HE4 ranked second with a sensitivity of 0.99 (95% CI 0.94, 1.0) and MK ranked third with a sensitivity of 0.91 (95% CI 0.79, 0.99). Several other proteins showed relatively high sensitivity values at 95% specificity, including KLK6, FR-alpha, and hk11.

**Table 2:** Comparison of Proseek^®^ values obtained from women with late stage ovarian cancer versus healthy women

Protein	Late versus healthy			
	AUC (95% CI)	Rank	Sensitivity at 95% specificity (95% CI)	Rank
CA.125	1 (1, 1)	1	1 (1, 1)	1
HE4	1 (0.99, 1)	2	0.99 (0.94, 1)	2
MK	0.98 (0.94, 1)	3	0.91 (0.79, 0.99)	3
KLK6	0.95 (0.9, 0.99)	4	0.88 (0.8, 0.96)	4
hK11	0.93 (0.88, 0.98)	5	0.79 (0.63, 0.93)	6
FR.alpha	0.92 (0.83, 0.98)	6	0.81 (0.67, 0.94)	5
CXCL13	0.92 (0.85, 0.97)	7	0.66 (0.43, 0.87)	7
IL.6	0.89 (0.8, 0.97)	8	0.59 (0.26, 0.85)	10
PDGF.subunit.B	0.87 (0.77, 0.95)	9	0.42 (0.2, 0.76)	19
U.PAR	0.86 (0.75, 0.95)	10	0.39 (0.12, 0.77)	23
FADD	0.84 (0.74, 0.93)	11	0.59 (0.36, 0.81)	9
TNFSF14	0.84 (0.72, 0.94)	12	0.38 (0.17, 0.71)	29
IL.7	0.84 (0.72, 0.93)	13	0.46 (0.25, 0.7)	16
CSF.1	0.84 (0.7, 0.95)	14	0.28 (0.04, 0.74)	39
CD40.L	0.83 (0.72, 0.95)	15	0.22 (0.02, 0.82)	53
TGF.alpha	0.83 (0.71, 0.93)	16	0.38 (0.14, 0.72)	30
PRSS8	0.83 (0.71, 0.93)	17	0.49 (0.3, 0.74)	15
MMP.1	0.82 (0.69, 0.92)	18	0.26 (0.08, 0.58)	47
FUR	0.82 (0.7, 0.92)	19	0.28 (0.09, 0.62)	40
TNF.R1	0.82 (0.69, 0.93)	20	0.44 (0.22, 0.72)	18
ILT.3	0.81 (0.67, 0.92)	21	0.39 (0.15, 0.68)	28
SCF	0.81 (0.72, 0.89)	22	0.64 (0.46, 0.8)	8
NTRK3	0.8 (0.67, 0.9)	23	0.53 (0.25, 0.76)	11
HGF	0.8 (0.66, 0.92)	24	0.38 (0.14, 0.67)	32
HB.EGF	0.8 (0.65, 0.93)	25	0.27 (0.06, 0.65)	42

The 25 proteins with the highest AUC values were ranked, as well as their sensitivity at 95% specificity. ROC curves for discriminating late stage high grade serous ovarian cancer versus healthy women for the 12 proteins with the highest AUC values are shown in Fig. 5. Data for all 92 proteins is provided in Additional file 2

Furthermore, ROC curve analyses were completed for each of the 92 proteins to determine which proteins could discriminate between sera from late stage ovarian cancer and women with benign ovarian disease. The 25 proteins with the highest AUC values are listed in Table 3, while the AUC values for all 92 proteins are listed in Additional file 3. The AUC for CA125 was 1.0 (95% CI 0.97, 1.0), which was the same as the AUC for HE4 1.0 (95% CI 0.98, 1.00). In total, 35 proteins had an estimated AUC of at least 0.70 and were significantly associated with cancer status. Again, many of the same proteins that were identified by unsupervised hierarchical clustering and by linear regression analysis were also significantly higher in the late stage ovarian cancer patients relative to the women with benign ovarian disease. The sensitivity at 95% specificity was also calculated and is shown for the top 25 proteins in Table 3, and for all 92 proteins in Additional file 3. CA125 had the highest sensitivity with a value of 1.0 (95% CI 0.89, 1.0), while HE4 ranked second with a sensitivity of 0.99 (95% CI 0.91, 1.0) and KLK6 ranked third with a sensitivity of 0.88 (95% CI 0.74, 0.97). Several other proteins showed relatively high sensitivity values at 95% specificity, including MK, FR-alpha, and hk11.

**Table 3:** Comparison of Proseek^®^ values obtained from women with late stage ovarian cancer versus benign disease

Protein	Late versus benign			
	AUC (95% CI)	Rank	Sensitivity at 95% specificity (95% CI)	Rank
HE4	1 (0.98, 1)	1	0.99 (0.91, 1)	2
CA.125	1 (0.97, 1)	2	1 (0.89, 1)	1
KLK6	0.95 (0.91, 0.99)	3	0.88 (0.74, 0.97)	3
MK	0.95 (0.9, 0.99)	4	0.79 (0.61, 0.96)	4
hK11	0.93 (0.87, 0.97)	5	0.7 (0.48, 0.91)	6
FR.alpha	0.91 (0.83, 0.98)	6	0.75 (0.55, 0.93)	5
IL.6	0.91 (0.82, 0.97)	7	0.69 (0.44, 0.88)	7
CSF.1	0.87 (0.75, 0.96)	8	0.4 (0.12, 0.8)	20
CXCL13	0.86 (0.77, 0.95)	9	0.6 (0.32, 0.82)	9
EZR	0.86 (0.76, 0.93)	10	0.63 (0.42, 0.83)	8
PRSS8	0.86 (0.74, 0.94)	11	0.56 (0.32, 0.81)	12
FUR	0.84 (0.74, 0.94)	12	0.48 (0.27, 0.76)	15
AM	0.83 (0.71, 0.94)	13	0.46 (0.22, 0.77)	17
CXCL10	0.83 (0.73, 0.92)	14	0.57 (0.32, 0.8)	10
TNF.R1	0.8 (0.67, 0.93)	15	0.36 (0.09, 0.75)	23
CSTB	0.79 (0.64, 0.92)	16	0.36 (0.11, 0.76)	22
ILT.3	0.79 (0.65, 0.91)	17	0.32 (0.09, 0.7)	26
CXCL9	0.78 (0.65, 0.9)	18	0.48 (0.21, 0.74)	16
VEGF.A	0.77 (0.65, 0.88)	19	0.52 (0.26, 0.71)	14
IL.7	0.76 (0.62, 0.89)	20	0.38 (0.14, 0.64)	21
ITGA1	0.76 (0.62, 0.88)	21	0.57 (0.38, 0.75)	11
ErbB4.HER4	0.75 (0.62, 0.86)	22	0.53 (0.32, 0.7)	13
IL.8	0.74 (0.56, 0.9)	23	0.2 (0.01, 0.66)	50
MCP.1	0.74 (0.59, 0.89)	24	0.22 (0.06, 0.57)	43
U.PAR	0.74 (0.57, 0.85)	25	0.15 (0.03, 0.41)	58

The 25 proteins with the highest AUC values were ranked for discriminating late stage high grade serous ovarian cancer versus benign ovarian disease. Their ranking for sensitivity at 95% specificity is also provided. Data for all 92 proteins is provided in Additional file 3

### ROC/AUC for individual proteins for early stage ovarian cancer

**Fig. 6:**
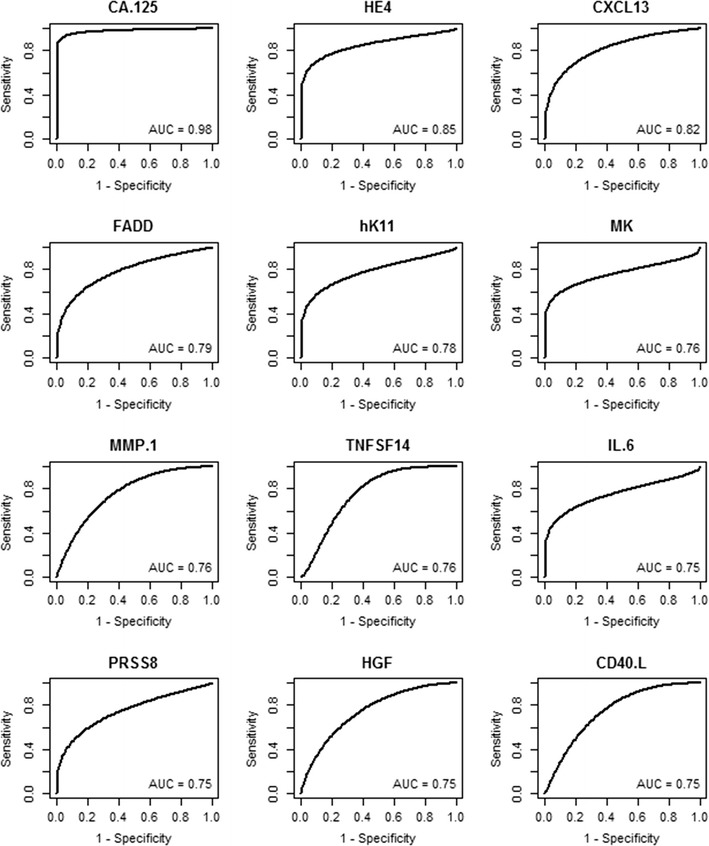
ROC curves for discriminating early stage ovarian cancer versus healthy women. ROC curves were graphed for the 12 proteins with the highest AUC values for discriminating early stage serous ovarian cancer versus healthy women. Data for the 25 proteins with the highest AUC values is shown in Table 4. Data for all 92 proteins is shown in Additional file 4

ROC curve analyses were completed for each of the 92 proteins to determine if there were proteins that could discriminate between early stage ovarian cancer samples and healthy women. ROC curves of the 12 proteins with the highest AUC values are graphed in Fig. 6. The 25 proteins with the highest AUC values are listed in Table 4, while the AUC values for all 92 proteins are listed in Additional file 4. The AUC for CA125 was 0.98 (95% CI 0.94, 1.0) and the AUC for HE4 was 0.85 (95% CI 0.74, 0.95) (Fig. 6). In total, 23 proteins had an estimated AUC of at least 0.7 and were significantly associated with cancer status, thus motivating further investigation. Many of the same proteins that were identified in the late stage ovarian cancer patients were also significantly higher in the early stage ovarian cancer patients relative to the healthy women (Fig. 6). However, several of the proteins that best discriminated between late stage versus healthy women (Fig. 5) did not discriminate between early stage versus healthy with an AUC < 0.7, e.g. PDGF-β, KLK6, and FR-alpha. The sensitivity at 95% specificity was also calculated and is shown for the top 25 proteins in Table 4, and for all 92 proteins in Additional file 4. CA125 had the highest sensitivity with a value of 0.93 (95% CI 0.81, 0.99), while HE4 ranked second with a sensitivity of 0.63 (95% CI 0.44, 0.84).

**Table 4:** Comparison of Proseek^®^ values obtained from women with early stage ovarian cancer versus healthy women

Protein	Early versus healthy			
	AUC (95% CI)	Rank	Sensitivity at 95% specificity (95% CI)	Rank
CA.125	0.98 (0.94, 1)	1	0.93 (0.81, 0.99)	1
HE4	0.85 (0.74, 0.95)	2	0.63 (0.44, 0.84)	2
CXCL13	0.82 (0.68, 0.93)	3	0.44 (0.19, 0.71)	7
FADD	0.79 (0.67, 0.9)	4	0.41 (0.14, 0.69)	10
hK11	0.78 (0.65, 0.88)	5	0.49 (0.26, 0.71)	4
MK	0.76 (0.65, 0.87)	6	0.53 (0.33, 0.72)	3
MMP.1	0.76 (0.62, 0.88)	7	0.19 (0.05, 0.49)	35
TNFSF14	0.76 (0.63, 0.89)	8	0.09 (0, 0.38)	65
IL.6	0.75 (0.65, 0.85)	9	0.47 (0.18, 0.68)	5
PRSS8	0.75 (0.62, 0.88)	10	0.37 (0.16, 0.59)	13
HGF	0.75 (0.6, 0.87)	11	0.21 (0.01, 0.51)	29
CD40.L	0.75 (0.6, 0.91)	12	0.15 (0, 0.52)	41
GDF.15	0.73 (0.58, 0.86)	13	0.41 (0.22, 0.63)	11
CD69	0.73 (0.59, 0.86)	14	0.18 (0.03, 0.5)	36
SCF	0.72 (0.59, 0.85)	15	0.39 (0.14, 0.63)	12
LAP.TGF.beta.1	0.72 (0.57, 0.86)	16	0.14 (0.02, 0.44)	44
U.PAR	0.72 (0.55, 0.86)	17	0.15 (0.02, 0.5)	42
NTRK3	0.71 (0.55, 0.86)	18	0.15 (0.02, 0.45)	40
Ep.CAM	0.71 (0.53, 0.87)	19	0.24 (0.09, 0.51)	21
TF	0.7 (0.55, 0.85)	20	0.19 (0.06, 0.44)	34
LITAF	0.7 (0.52, 0.86)	21	0.03 (0, 0.39)	81
CDH3	0.7 (0.56, 0.88)	22	0.42 (0.22, 0.67)	8
EGFR	0.7 (0.54, 0.85)	23	0.08 (0.01, 0.37)	66
VEGF.A	0.69 (0.53, 0.83)	24	0.21 (0.05, 0.5)	28
TGF.alpha	0.69 (0.53, 0.84)	25	0.09 (0.01, 0.36)	62

The 25 proteins with the highest AUC values were ranked, as well as their sensitivity at 95% specificity. ROC curves for discriminating sera from early stage high grade serous ovarian cancer versus healthy women for the 12 proteins with the highest AUC values are shown in Fig. 6. Data for all 92 proteins is provided in Additional file 4

ROC curve analyses were then completed for each of the 92 proteins to determine if the proteins could discriminate between sera from women with early stage ovarian cancer versus benign ovarian disease. The 25 proteins with the highest AUC values are listed in Table 5, while the AUC values for all 92 proteins are listed in Additional file 5. The AUC for CA125 was 0.87 (95% CI 0.73, 0.97) and the AUC for HE4 was 0.87 (95% CI 0.76, 0.96). In total, 10 proteins had an estimated AUC of at least 0.7 and were significantly associated with cancer status, including hK11, PRSS8, IL-6, MK, and CXCL13. The sensitivity at 95% specificity was also calculated and is shown for the top 25 proteins in Table 5, and for all 92 proteins in Additional file 5. Few proteins exhibited adequate sensitivity for the discrimination of early stage ovarian cancer from benign disease; HE4 showed the highest sensitivity of 0.60 (95% CI 0.37, 0.88).

**Table 5:** Comparison of Proseek^®^ values obtained from women with early stage ovarian cancer versus benign disease

Protein	Early versus benign			
	AUC (95% CI)	Rank	Sensitivity at 95% specificity (95% CI)	Rank
HE4	0.87 (0.76, 0.96)	1	0.6 (0.37, 0.88)	1
CA.125	0.87 (0.73, 0.97)	2	0.46 (0.13, 0.93)	3
hK11	0.79 (0.67, 0.89)	3	0.38 (0.15, 0.65)	9
PRSS8	0.78 (0.64, 0.89)	4	0.44 (0.2, 0.7)	4
IL.6	0.75 (0.65, 0.86)	5	0.54 (0.28, 0.7)	2
MK	0.72 (0.57, 0.85)	6	0.37 (0.13, 0.61)	11
CXCL13	0.71 (0.53, 0.85)	7	0.39 (0.16, 0.66)	7
CXCL10	0.71 (0.57, 0.85)	8	0.35 (0.14, 0.62)	13
EZR	0.7 (0.55, 0.83)	9	0.33 (0.11, 0.56)	14
CSTB	0.7 (0.53, 0.85)	10	0.21 (0.01, 0.62)	23
FR.alpha	0.69 (0.56, 0.81)	11	0.39 (0.08, 0.62)	8
VEGF.A	0.69 (0.52, 0.84)	12	0.37 (0.19, 0.6)	10
KLK6	0.69 (0.54, 0.8)	13	0.41 (0.11, 0.63)	5
FUR	0.68 (0.53, 0.83)	14	0.16 (0.04, 0.42)	34
CSF.1	0.68 (0.5, 0.83)	15	0.14 (0.01, 0.41)	42
AM	0.68 (0.5, 0.82)	16	0.26 (0.07, 0.54)	18
LYN	0.67 (0.56, 0.79)	17	0.39 (0.05, 0.59)	6
ICOSLG	0.67 (0.51, 0.82)	18	0.19 (0.02, 0.47)	28
CDH3	0.67 (0.51, 0.83)	19	0.35 (0.21, 0.6)	12
CXCL9	0.66 (0.49, 0.82)	20	0.28 (0.09, 0.57)	16
ILT.3	0.66 (0.5, 0.82)	21	0.17 (0.02, 0.49)	32
GDF.15	0.66 (0.49, 0.84)	22	0.1 (0, 0.54)	51
IFN.gamma	0.65 (0.5, 0.79)	23	0.26 (0.01, 0.55)	17
EGFR	0.65 (0.49, 0.8)	24	0.1 (0.01, 0.37)	56
MCP.1	0.65 (0.48, 0.81)	25	0.09 (0, 0.36)	58

The 25 proteins with the highest AUC values were ranked for discriminating early stage high grade serous ovarian cancer versus benign ovarian disease. Their ranking for sensitivity at 95% specificity is also provided. Data for all 92 proteins is provided in Additional file 5

### Combinations of biomarkers

**Fig. 7:**
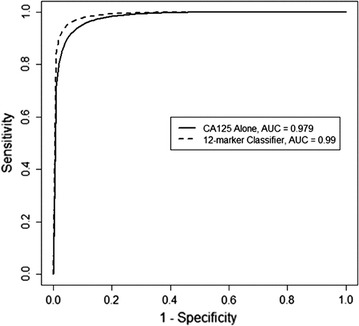
ROC curves for CA125 alone and the 12-protein classifier. ROC curves were graphed for discriminating early stage serous ovarian cancer versus healthy women for CA125 alone (solid line; AUC = 0.979) and the 12-protein classifier (dashed line; AUC = 0.99) developed using supervised machine learning techniques

In addition to evaluating single proteins, we also used machine learning techniques to develop a multi-biomarker model for discriminating between early stage ovarian cancer cases versus healthy women. Using a naïve Bayes classifier that combined 12 proteins, we improved the AUC from 0.979 to 0.99 when compared to CA125 alone (Fig. 7), and the sensitivity corresponding to 95% specificity increased from 93 to 95.2% when compared to CA125 alone. The 12 proteins in the multi-biomarker model were: CA125, CD40.L, CD69, CXCL9, CXCL13, epidermal growth factor receptor (EGFR), epithelial cell adhesion molecule (EpCAM), protein deglycase DJ-1 (also known as Parkinson disease protein 7) (PARK7), E-selectin (SELE), latency-associated peptide transforming growth factor-β1 (LAP.TGF.beta.1), tissue factor (TF), and VEGFR2.

## Discussion

This is the first study to use the Proseek^®^ multiplex Oncology I v2 plate to quantify 92 cancer-related proteins simultaneously in sera from ovarian cancer patients. By using two distinct clustering methods (PCA and unsupervised hierarchical clustering), we could separate the healthy/benign samples from the early/late stage serous ovarian cancer samples, suggesting a multiplex assay may be more robust than any single protein measurement at identifying ovarian cancer. The CA125 values that were obtained on the Proseek^®^ plates correlated with the clinical laboratory ELISA values, suggesting that the PEA technology used in the Proseek^®^ plates provides a comparable means of analysis of serum samples. Previous studies comparing ELISA values to PEA for biomarkers of colorectal cancer (CEA, IL-8, TIMP-1 and CA242) found correlations ranging from 0.73 to 0.98 [24, 42]. In addition, Fredriksson et al. [53] evaluated a multiplex proximity ligation assay (PLA), a precursor to PEA technology, for 20 biomarkers in plasma from 19 ovarian cancer patients of different histological subtypes and stages. A direct comparison between the CA125 values from the PLA versus a Luminex assay resulted in a good correlation between the two assays.

We conducted comprehensive literature searches to determine which of the 92 proteins present on the Proseek^®^ Multiplex Oncology I v2 plate have been linked to ovarian cancer. Over half (53/92) of the oncology proteins were reported to have increased expression in ovarian cancer; however, the presence of many of these 53 proteins had only been examined in ovarian cancer tissues, not in serum samples. Only ~ 10% of the 92 proteins were reported to have lower levels of expression in ovarian cancer than control samples. The remaining 32 oncology-related proteins on the Oncology Iv2 plate have yet to be investigated for their expression levels in ovarian cancer biospecimens (tissue or sera).

We identified many proteins on the Proseek^®^ plate that positively correlated with ovarian cancer. Overall, statistically significant (p < 0.001) trends were observed for 38 of the 92 proteins, many of which have yet to be documented as candidate serum biomarkers for ovarian cancer. The 12 most significant proteins were: CA125, HE4, MK, KLK6, hK11, CXCL13, FR-alpha, IL-6, TNFSF14, FADD, PRSS8, and FUR. Eight of these twelve proteins have previously been described as having elevated levels in sera from ovarian cancer patients, while FUR was previously identified in ovarian cancer tumor tissues and cell lines, and was associated with decreased survival [54, 55]. Notably, CXCL13, TNFSF14, and FADD had not been previously identified in ovarian cancer tissues or serum.

Mucin 16 (MUC16), more commonly referred to as CA125, is the biomarker primarily used to monitor ovarian cancer recurrence and for differential diagnosis of pelvic masses [8, 56, 57]. The high AUC values that we obtained on the Proseek^®^ plates (0.88–1.0) comparing sera from early and late stage serous ovarian cancer patients to sera from healthy or benign ovarian disease, confirm the extensive literature of the past two decades on the usefulness of CA125 as an ovarian cancer biomarker [7–9]. With a specificity of 95%, CA125 achieved 100% sensitivity on the Proseek^®^ plates when comparing sera from late stage serous ovarian cancer cases versus healthy women or women with benign ovarian disease, and 93% sensitivity when comparing sera from early stage serous ovarian cancer cases versus healthy women. However, the sensitivity for CA125 decreased dramatically to 46% when comparing sera from early stage serous ovarian cancer cases versus women with benign ovarian disease.

Our Proseek^®^ results are in agreement with previous studies [58, 59], as we found that HE4 consistently performed very closely to CA125, with AUC values of 0.85–1.0 when comparing sera from early and late stage serous ovarian cancer patients to sera from healthy women or women with benign ovarian disease. With a specificity of 95%, HE4 achieved 99% sensitivity on the Proseek^®^ plates when comparing sera from late stage ovarian cancer cases to healthy women or women with benign ovarian disease. However, the sensitivity for HE4 decreased to ~ 60% when comparing sera from early stage ovarian cancer cases to healthy women or women with benign ovarian disease.

In addition to the well-known ovarian cancer serum biomarkers CA125 and HE4, we identified six additional proteins, MK, hK11, KLK6, FRα, PRSS8, and IL-6, that had previously been reported as serum biomarkers for ovarian cancer. High expression levels of MK have been found in both the serum and tissue of patients with ovarian cancer [33]. Rice et al. [60] reported that when MK was used as a single biomarker, the AUC (0.734) was not as good as CA125; however, when MK was combined in a multi-analyte panel including CA125 and anterior gradient 2 protein, the sensitivity and specificity improved. The AUC values that we obtained on the Proseek^®^ plate were 0.98 when comparing late stage ovarian cancer to healthy women, and 0.95 when comparing late stage ovarian cancer to women with benign disease. However, the AUC values decreased significantly to 0.76 and 0.72 when comparing early stage ovarian cancer to healthy women or women with benign disease, respectively. In addition, the sensitivity of MK was high (91%) when comparing late stage ovarian cancer to healthy women, but not in other comparisons.

Human kallikrein 11 (hK11) has also been validated as a serum biomarker for ovarian cancer, alone and in combination with CA125 [61]. Similar to our studies, McIntosh et al. [61] found hK11 was less sensitive than CA125 at detecting ovarian cancer versus healthy controls, although hK11 was among the top five most sensitive individual proteins in our study. We observed only slight differences in sensitivity when comparing cancer to healthy and cancer to benign samples, while McIntosh et al. [61] found the sensitivity of hK11 was substantially decreased when comparing cancer versus benign samples.

Elevated KLK6 levels have been associated with late stage ovarian cancer but not benign tumors [34]. A recent study using western blots of serum depleted of high abundance proteins suggests that serum KLK6 levels are elevated in early stages of serous ovarian cancer [62]. Analyses of our Proseek^®^ data support these findings of a high correlation between KLK6 serum levels and late stage ovarian cancer. By linear regression, we also found that serum levels of KLK6 were nearly identical for healthy women and women with benign ovarian disease. We found the specificity of KLK6 to be relatively high (88%) when comparing late stage ovarian cancer to healthy or benign cases; however the sensitivity was much lower (~ 40%) when comparing early stage ovarian cancer to healthy or benign cases.

FRα has also previously been tested as a biomarker for ovarian cancer detection. Using an electrochemiluminescent assay, O’Shaunessy et al. [36] showed that as a single biomarker, FRα was able to discriminate between ovarian cancer and normal serum samples, with AUCs ranging from 0.62 for stage I samples to 0.94 and 0.92 for stage III and IV samples [36]. We found similar results using the Proseek^®^ assay for detection of FRα alone; the AUC was 0.92 for late stage ovarian cancer versus healthy controls and 0.69 for detection of early stage ovarian cancer cases versus healthy controls. Another study used an ELISA for the detection of FRα in patients undergoing surgery for a pelvic mass [63]. In their study, FRα was slightly better than CA125 for discriminating between malignant and benign ovarian masses. Our Proseek^®^ data indicate that FRα expression is not impacted by benign ovarian disease, since we observed similar data for healthy and benign cases (Fig. 4) and when computing the AUC and sensitivity values.

Mok et al. [64] demonstrated an upregulation of human prostasin (PRSS8) in ovarian cancer cells compared to normal cells, and increased levels of PRSS8 in sera from ovarian cancer patients compared to normal controls. Recent western blot analysis of sera that had been depleted of the highly abundant proteins showed that PRSS8 levels were increased in early stage ovarian cancer samples compared to benign samples or healthy controls [65]. Our Proseek^®^ data corroborated these results as we found that PRSS8 is noticeably higher in the sera of women with early and late stage ovarian cancer as compared to healthy women or women with benign ovarian disease. The AUC values that we obtained in the various comparisons were relatively high (ranging from 0.75 in early stage to 0.86 in late stage); however the sensitivity was relatively low (ranging from 37% in early stage to 56% in late stage).

Our results support earlier studies which showed that serum IL-6 levels positively correlate with the clinical disease status of women with ovarian cancer [39, 66, 67]. The AUC values that we obtained in the various comparisons were relatively high (ranging from 0.75 in early stage to 0.91 in late stage); however the sensitivity was relatively low (ranging from 47% in early stage to 69% in late stage). A recent study of 14 cytokines in the plasma of ovarian cancer patients showed that IL-6 was the only one that was significantly elevated in patients with early stage ovarian cancer relative to benign disease [68]. However, when tested for the potential to improve the discriminatory capability of CA125, IL-6 levels did not contribute significantly [68].

Although the majority of the proteins we identified with the Proseek^®^ plates had previously been investigated as serum biomarkers for ovarian cancer, several of the proteins that we identified are novel serum biomarkers for ovarian cancer. Increased expression of the serine protease Furin (FUR) was shown by immunohistochemistry in primary ovarian tumor tissues and metastases, and was associated with decreased survival [55]. Our data extends these studies by showing that FUR can also be detected in sera of women with ovarian cancer. The AUC values that we obtained in the various comparisons were moderate (ranging from 0.68 in early stage to 0.84 in late stage).

Several biomarkers that showed high serum levels in the Proseek^®^ plate have not been previously examined in ovarian cancer samples. Expression of the CXC chemokine 13 (CXCL13) has been reported in tumors and serum of breast cancer [69], non-small cell lung carcinoma [70], and hepatocellular carcinoma [71], but little is currently known about the expression of CXCL13 in ovarian cancer. Our analysis shows that CXCL13 levels were significantly elevated in the sera of women with both early and late stage ovarian cancer, and the AUC values ranged from 0.71 (early stage) to 0.92 (late stage) when comparing cancer to healthy or benign samples.

Tumor necrosis factor superfamily member 14 (TNFSF14), also known as LIGHT, is a member of the tumor necrosis factor (TNF) ligand family involved in inflammatory disorders [72]. Little research currently exists for TNSFS14 expression in ovarian cancer, however, in metastatic colorectal cancer, increased LIGHT expression is associated with an increase in the number of tumor infiltrating T-cells and survival [73, 74]. We identified TNFSF14 as a potential biomarker for serous ovarian cancer due to its significantly higher levels in late stage ovarian cancer compared to its levels in serum samples from the other three groups we examined.

FAS-associated death domain (FADD) is an adaptor molecule that mediates cell apoptotic signals. FADD overexpression occurs frequently in squamous cell carcinoma of the head and neck, and is associated with metastasis [75]. Decreased cellular FADD levels have been found in non-small cell lung cancer, and have been associated with FADD release into the extracellular space [76]. Although FADD release could portend the detection of FADD in the serum, this has not been examined in other studies. Our results show that FADD levels are increased in the sera of women with ovarian cancer, with highest levels of FADD detected in late stage ovarian cancer.

Over the years, many studies have been conducted using newly discovered biomarkers for ovarian cancer [5, 7]. In most cases, the sensitivity and specificity of the biomarker by itself is reported, along with a ROC curve, to show that the AUC increases when the new biomarker is combined with CA125, as we have published for the proteins nectin-4 [17] and leucine-rich alpha-2-glycoprotein-1 [14]. Due to the low prevalence of ovarian cancer, a screening test must achieve a minimum specificity of 99.6% and a sensitivity of > 75% for early stage disease to avoid an unacceptable level of false-positive results and achieve a positive predictive value of 10% [77, 78].

In this study, we attempted to develop a classifier for early stage serous ovarian cancer compared to healthy women using multiple proteins from the Proseek^®^ plate. A naïve Bayes classifier that combined 12 proteins improved the AUC from 0.979 to 0.99 when compared to CA125 alone, and improved the sensitivity corresponding to 95% specificity from 93 to 95.2%. While this represents only an incremental improvement, these incremental improvements are required in the setting of population screening. Furthermore, our small sample size (~ 21 cases per group), particularly of early stage ovarian cancer cases, limited our ability to develop and validate a multi-biomarker model. The 12 proteins in our model were: CA125, CD40.L, CD69, CXCL9, CXCL13, EGFR, EpCAM, PARK7, SELE, LAP.TGF-β1, TF, and VEGFR2. Linear regression analysis of our Proseek^®^ data had found six of these proteins to have a statistically significantly trend (p < 0.001); CA125, CXCL13, CD40L, CD69, and LAP.TGF-β1 levels were higher in ovarian cancer serum samples, while EGFR levels were lower. Three other proteins (TF, VEGFR, and CXCL9) also showed a lower, albeit significant trend. When the classification accuracy of our Proseek^®^ data was evaluated using the ROC curve, 8 of the 12 proteins in our multi-protein classifier had AUC values at or above 0.7 (CA125, CXCL13, CD40L, CD69, LAP-TGF-β1, EpCAM, TF, and EGFR) when tested individually; the levels of three of these proteins (EpCAM, TF, and EFGR) were lower in the early stage ovarian cancer serum samples relative to the healthy controls. Except for CA125 with a sensitivity of 93% at 95% specificity, none of the other proteins had a sensitivity greater than 0.63 when tested individually. Of the 11 proteins that were added to CA125 in the multi-protein classifier, eight of the proteins (CD40.L, CD69, CXCL9, EGFR, EpCAM, SELE, TF, and VEGFR2) have been associated with ovarian cancer in previous studies [29, 79–84]; however, CXCL9 has only been examined in ovarian cancer tissues [85], and CD69 was associated with T-lymphocytes in ovarian cancer ascites fluid [86]. The remaining three proteins (CXCL13, PARK7, and LAP.TGF-β1) do not appear in the literature as having an association with ovarian cancer, however they have been identified in other types of cancer [69–71, 87, 88]. Interestingly, 6 of the 11 proteins that were added to CA125 in the multi-protein classifier were lower in ovarian cancer sera relative to the healthy controls, suggesting that future biomarker discovery studies should not exclusively focus on proteins that have increased levels in ovarian cancer serum.

The multivariable technique is currently used in the FDA-cleared OVA1^®^ qualitative serum test, in which the serum levels of five proteins [prealbumin (also called transthyretin), apolipoprotein A-1, β2 microglobulin, transferrin, and CA 125 II] [89–92] are determined by five immunoassays. The OVA1^®^ score in combination with the menopausal status of the woman, is used to determine whether a woman with an adnexal mass has a low or high probability of malignancy. The OVA1^®^ test “is not intended as a screening or stand-alone diagnostic assay” [93].

Yurkovetsky et al. [94] used a Metropolis algorithm with Monte Carlo simulation to analyze 96 serum biomarkers on the multiplex xMAP bead-based immunoassays (Luminex). They were able to identify an optimal biomarker panel comprised of four biomarkers (CA-125, HE4, CEA, and VCAM-1) that discriminated early stage ovarian cancer from healthy controls, with a specificity of 98% and a sensitivity of 86%. As mentioned above, this level of specificity is still not sufficient to screen the general population.

The reproducibility of the PEA technology used in the Proseek^®^ plates has been documented by the manufacturer (http://www.olink.com) and others [22–24] in which technical replicates were used to evaluate intra-assay and inter-assay variation. These reports successively incorporated technical improvements to the assay technology which have increased its reproducibility. Using the same assay protocol that was used for our experiments, Assarsson et al. [22] measured the levels of 92 cancer biomarkers with an average inter-assay coefficient of variation (CV) of 12%. Using the precision statistics available from the Olink website, the average CV for the 12 proteins that comprise our multi-protein classifier ranged from 13 to 22% (average CV of 18.9%). This is similar to values obtained using bead-based assays of inflammatory markers [21] and in bead- or plate-based assays of ovarian cancer biomarkers, in which the CV ranged from 12 to 25% [95].

In this study, we focused on high grade serous ovarian cancer, since it is the most prevalent and deadly subtype of ovarian cancer [45]. Although the serous ovarian cancer subtype comprises approximately 80% of ovarian cancers, it is possible that the results that we obtained will not translate to the other 20% of ovarian cancer subtypes (mucinous, clear cell, endometrioid, mixed Mullerian). It will be necessary for us to perform additional studies using serum samples from the other ovarian cancer subtypes in order to determine whether they can correctly be classified by our multi-protein classifier. It is possible that we may need to add more proteins to our multi-protein classifier in order to successfully classify all of the major ovarian cancer subtypes, since serum levels of CA125 are typically highest in women with late stage serous ovarian cancer compared to the other ovarian cancer subtypes. Escudero et al. [96] reported that the serum levels of CA125 and HE4 are highest in the serous subtype of ovarian cancer (over 84% positive) and lowest in the mucinous subtype (68.5 and 43.8% positive, respectively). Similarly, Kristjansdottir et al. [97] reported that CA125 levels were highest in serous (297 U/ml), followed by clear cell (194 U/ml) and endometrioid (132 U/ml), and lowest in mucinous (36 U/ml). They also reported that serum levels of HE4 were highest in the serous and endometrioid subtypes and low in mucinous and clear cell subtypes [97]. Likewise, Hertlein et al. [98] reported that median HE4 levels were over five-fold higher in serous compared to mucinous ovarian cancer. Other serum markers such as CA 19.9 or REG4 that have been reported to be elevated in the sera of mucinous ovarian cancer patients [99, 100] may need to be incorporated into our next generation of multi-protein classifier.

Our future studies will focus on refining our model and validating our results by using a large number of asymptomatic samples from other sources (e.g. PLCO and UKCTOCS) in which serum samples were obtained several years prior to the diagnosis of high grade serous ovarian cancer [12, 101, 102]. Others have tested dozens of biomarkers individually on different platforms at different sites for their ability to discriminate between ovarian cancer cases (symptomatic and asymptomatic cases) versus benign disease, and CA125 was found to be the single best biomarker [95]. Even when six to eight biomarkers were combined into one test, there was no improvement over CA125 alone [103]. Only a handful of biomarkers (including CA125) have been tested to date using techniques other than ELISA; none of the biomarkers were an improvement upon CA125 [104]. It may be that the biomarkers that were tested had all been discovered through the use of late stage diagnostic specimens, as suggested by Zhu et al. [103], and thus, those biomarkers may not have been the optimal choices for screening early stages of disease.

Upon validation of our results with a larger cohort of patients, we envision collaborating with Olink to design multiplex plates that will contain a small subset of the 92 markers, encompassing the most relevant proteins that are capable of distinguishing women with ovarian cancer from healthy women. Ideally, the multiplex plates would be limited to a small number of proteins that would allow us to achieve a high degree of sensitivity and specificity. Since the PEA technology combines the sensitivity of the PCR with the specificity of antibody-based detection methods, it may prove to be readily translated into clinical laboratories by virtue of its ability to perform multiplex biomarker detection and high throughput quantification. Other technologies, such as ELISAs are limited in their ability to adequately detect multiple biomarkers simultaneously. In addition, mass spectrometry based assays are not routinely performed in clinical laboratories due to the high cost of instrumentation and technical staffing.

Although the Proseek^®^ Oncology Iv2 plate was not designed specifically for ovarian cancer, it included several of the key proteins known to be elevated in ovarian cancer (e.g. CA125 and HE4). A customized plate targeting ovarian cancer proteins could enhance the ability of this technology to identify ovarian cancer samples. Using a multiplex approach, rather than just one biomarker at a time, it may be possible to: (a) diagnose ovarian cancer in a woman with an abdominal mass prior to surgery, (b) screen high risk women for ovarian cancer, and (c) ultimately screen the general population of women for ovarian cancer. Our goal is to detect ovarian cancer in its earliest stages, when the survival rate is > 90% [105]. Earlier detection will enhance the treatment options for women, since women who are diagnosed early require less extensive surgery and less toxic chemotherapy.

## Conclusions

A biomarker panel that can be used to screen the sera of women in the general population for ovarian cancer is needed to detect early stages of ovarian cancer, when the disease is most amenable to treatment and the survival rates are highest. In this study, we have shown the feasibility of using Proseek^®^ multiplex Oncology I v2 plates to quantify the levels of 92 oncology-related proteins in only 1 μl of sera. These data demonstrate that the Proseek^®^ technology can replicate the results established by conventional clinical assays for known biomarkers, such as CA125 and HE4, and identify new candidate biomarkers for ovarian cancer. A multi-protein classifier consisting of CA125 and eleven other proteins was able to improve the sensitivity and specificity over CA125 alone. Additional studies using a larger cohort of patients will allow for validation of these biomarkers and lead to the development of a screening tool for detecting early stage ovarian cancer in the general population.

## Additional files

**Additional file 1.** List of all 92 proteins with their significance in trends among patient subgroups. List of the 92 Proseek^®^ Oncology I v2 proteins in order of significance showing a trend of log values when comparing serum samples of healthy women versus benign ovarian disease versus early stage serous ovarian cancer versus late stage serous ovarian cancer. The log data for the top 12 proteins are graphed in Fig. 4.

**Additional file 2.** AUC and sensitivity for all 92 proteins comparing late stage ovarian cancer versus healthy. Comparison of Proseek^®^ Oncology I values for serum samples from late stage high grade serous ovarian cancer patients versus healthy women. ROC curves for the 12 proteins with the highest AUC values are shown in Fig. 5.

**Additional file 3.** AUC and sensitivity for all 92 proteins comparing late stage ovarian cancer versus benign. Comparison of Proseek^®^ Oncology I values for serum samples from late stage high grade serous ovarian cancer patients versus women with benign ovarian conditions.

**Additional file 4.** AUC and sensitivity for all 92 proteins comparing early stage ovarian cancer versus healthy. Comparison of Proseek^®^ Oncology I values for serum samples from early stage high grade serous ovarian cancer patients versus healthy women. ROC curves for the 12 proteins with the highest AUC values are shown in Fig. 6.

**Additional file 5.** AUC and sensitivity for all 92 proteins comparing early stage ovarian cancer versus benign. Comparison of Proseek^®^ Oncology I values for serum samples from early stage high grade serous ovarian cancer patients versus women with benign ovarian conditions.

## Abbreviations

AUC: area under the ROC curve
CA125: cancer antigen 125
CEA: carcinoembryonic antigen
CI: confidence interval
C_t_: cycle threshold
CV: coefficient of variation
CXCL13: CXC motif chemokine 13
ELISA: enzyme-linked immunosorbent assay
EGFR: epidermal growth factor receptor
EpCAM: epithelial cell adhesion molecule
FADD: FAS-associated death domain protein
FDA: Food and Drug Administration
FR-alpha: folate receptor-alpha
FUR: furin
HE4: human epididymis protein 4
hK11: human kallikrein 11
IL-6: interleukin-6
KLK6: kallikrein 6
LAP.TGF-beta 1: Latency associated peptide transforming growth factor-β1
MK: midkine
MUC16: mucin 16
PARK7: protein deglycase DJ-1 (also known as Parkinson disease protein 7)
PCA: principle component analysis
PCR: polymerase chain reaction
PEA: proximity extension assay
PLA: proximity ligation assay
PLCO: Prostate, Lung, Colorectal, and Ovarian Cancer Screening Trial
PRSS8: prostasin
ROC: receiver operating characteristic
SELE: E-selectin
TF: tissue factor
TNF: tumor necrosis factor
TNFSF14: tumor necrosis factor ligand superfamily member 14
UKCTOCS: United Kingdom Collaborative Trial of Ovarian Cancer Screening
