# Supplementary material for: A multiplex platform for the identification of ovarian cancer biomarkers
Source: Clin Proteomics. 2017 Oct 10;14:34. doi: 10.1186/s12014-017-9169-6 (PMC5634875; doi:10.1186/s12014-017-9169-6)
Supplement: Supplementary file 1 — Additional file 1. List of all 92 proteins with their significance in trends among patient subgroups. List of the 92 Proseek® Oncology I v2 proteins in order of significance showing a trend of log values when comparing serum samples of healthy women versus benign ovarian disease versus early stage serous ovarian cancer versus late stage serous ovarian cancer. The log data for the top 12 proteins are graphed in Fig. 4. [file 12014_2017_9169_MOESM1_ESM.pdf]

Additional file 1.

Protein	Healthy	Benign ovarian disease	Early stage high grade serous ovarian cancer	Late stage high grade serous ovarian cancer	p-value
CA.125	2.31 (0.64)	3.14 (1.46)	5.39 (1.37)	7.25 (0.44)	< 0.001
HE4	7.7 (0.33)	7.56 (0.46)	8.48 (0.67)	9.22 (0.38)	< 0.001
MK	6.82 (0.42)	6.91 (0.62)	7.59 (0.99)	8.55 (0.77)	< 0.001
KLK6	5.33 (0.36)	5.26 (0.42)	5.74 (0.89)	7.13 (1.02)	< 0.001
hK11	5.78 (0.44)	5.67 (0.64)	6.48 (0.79)	7.13 (0.78)	< 0.001
CXCL13	8.39 (0.5)	8.72 (0.35)	9.12 (0.63)	9.45 (0.59)	< 0.001
FR.alpha	5.42 (0.35)	5.37 (0.5)	5.91 (0.96)	6.85 (0.98)	< 0.001
IL.6	4.26 (1.07)	4.34 (0.82)	5.88 (2.11)	6.29 (1.22)	< 0.001
TNFSF14	2.73 (0.92)	3.45 (0.65)	3.49 (0.55)	3.99 (0.88)	< 0.001
FADD	1.55 (0.4)	1.95 (0.42)	2.08 (0.53)	2.37 (0.71)	< 0.001
PRSS8	9.32 (0.36)	9.26 (0.34)	9.74 (0.51)	9.9 (0.5)	< 0.001
FUR	7.77 (0.52)	7.82 (0.34)	8.05 (0.34)	8.37 (0.43)	< 0.001
U.PAR	10.3 (0.32)	10.49 (0.33)	10.54 (0.27)	10.76 (0.27)	< 0.001
CSF.1	8.95 (0.28)	8.93 (0.25)	9.09 (0.23)	9.29 (0.2)	< 0.001
CD40.L	7.77 (1.16)	8.78 (0.45)	8.74 (0.89)	9.1 (0.74)	< 0.001
MMP.1	8.76 (1.21)	9.55 (0.83)	9.86 (0.99)	10.15 (0.92)	< 0.001
NTRK3	7.65 (0.31)	7.49 (0.29)	7.43 (0.27)	7.11 (0.55)	< 0.001
IL.7	2.83 (0.54)	3.1 (0.46)	3.26 (0.71)	3.66 (0.64)	< 0.001
ITGA1	7.2 (0.24)	7.11 (0.15)	7.08 (0.25)	6.8 (0.43)	< 0.001
ILT.3	2.88 (0.6)	2.92 (0.65)	3.3 (0.7)	3.67 (0.68)	< 0.001
HGF	7.64 (0.56)	7.98 (0.56)	8.14 (0.51)	8.36 (0.65)	< 0.001
TNF.R1	12.24 (0.32)	12.25 (0.37)	12.42 (0.42)	12.71 (0.41)	< 0.001
SCF	8.35 (0.29)	8.14 (0.7)	8 (0.5)	7.56 (0.86)	< 0.001
VEGF.A	10.54 (0.59)	10.63 (0.35)	10.99 (0.65)	11.23 (0.75)	< 0.001
PDGF.subunit.B	7.4 (0.5)	7.83 (0.45)	7.72 (0.7)	8.14 (0.42)	< 0.001
EZR	4.2 (0.31)	4.08 (0.23)	4.3 (0.35)	4.6 (0.43)	< 0.001
CD69	7.03 (0.75)	7.51 (0.61)	7.65 (0.68)	7.9 (0.82)	< 0.001
TGF.alpha	3.21 (0.76)	3.93 (0.81)	3.68 (0.57)	4.22 (0.76)	< 0.001
AM	6.64 (0.46)	6.48 (0.5)	6.87 (0.68)	7.25 (0.61)	< 0.001
LITAF	1.33 (1.04)	1.91 (0.8)	1.96 (0.58)	2.31 (0.82)	< 0.001
CSTB	6.14 (0.54)	6.03 (0.54)	6.45 (0.59)	6.7 (0.63)	< 0.001
CXCL11	4.84 (0.99)	5.22 (1.18)	5.38 (1.02)	6.02 (1.05)	< 0.001
VIM	5.09 (0.89)	5.44 (0.72)	5.48 (0.61)	5.88 (0.47)	< 0.001
CXCL10	7.73 (1.02)	7.36 (0.72)	8.11 (1.14)	8.78 (1.29)	< 0.001
LAP.TGF.beta.1	6.87 (0.59)	7.39 (0.45)	7.32 (0.48)	7.48 (0.47)	< 0.001
EGFR	4.87 (0.38)	4.79 (0.31)	4.63 (0.27)	4.52 (0.42)	< 0.001
HB.EGF	9.31 (0.77)	9.79 (0.64)	9.63 (0.73)	10.17 (0.69)	< 0.001
IL.8	6.96 (0.54)	6.88 (1.15)	7.31 (0.95)	7.89 (1.04)	< 0.001
CDH3	2.59 (0.35)	2.65 (0.39)	3.01 (0.74)	3.07 (0.59)	0.001
GDF.15	8.7 (0.46)	8.79 (0.9)	9.27 (0.79)	9.3 (0.58)	0.001
ICOSLG	4.86 (0.24)	4.84 (0.22)	4.69 (0.24)	4.58 (0.47)	0.002
LYN	-0.09 (0.31)	-0.05 (0.26)	0.22 (0.55)	0.21 (0.32)	0.003
TRAIL.R2	3.99 (0.38)	3.94 (0.61)	4.21 (0.52)	4.45 (0.61)	0.003
TNF.R2	5.12 (0.39)	5.12 (0.51)	5.3 (0.62)	5.61 (0.62)	0.003
PTPN22	3.28 (1.14)	3.79 (0.86)	3.68 (0.71)	4.18 (0.75)	0.003
TF	5.62 (0.35)	5.3 (0.32)	5.36 (0.35)	5.22 (0.55)	0.004

BAFF	6.63 (0.42)	6.7 (0.64)	6.74 (0.42)	7.09 (0.55)	0.006
FS	5.05 (0.56)	5.06 (0.52)	5.31 (0.53)	5.45 (0.5)	0.006
IL.1ra	6.16 (0.91)	6.33 (0.66)	6.57 (0.67)	6.74 (0.7)	0.007
FasL	2.26 (0.63)	2.14 (0.51)	1.99 (0.42)	1.85 (0.48)	0.007
ErbB4.HER4	5.97 (0.22)	5.87 (0.25)	6.03 (0.53)	6.32 (0.62)	0.008
CCL19	9.95 (0.79)	10.18 (1.04)	10.43 (0.89)	10.63 (1.03)	0.014
IL.17RB	2.23 (0.56)	2.42 (0.58)	2.45 (0.37)	2.66 (0.73)	0.021
VEGFR.2	8.13 (0.28)	8.11 (0.39)	8 (0.25)	7.93 (0.33)	0.023
AR	2.31 (0.55)	2.46 (0.6)	2.66 (0.46)	2.7 (0.88)	0.033
CASP.3	7.91 (0.7)	7.98 (0.57)	8.2 (0.71)	8.35 (0.89)	0.033
MCP.1	10.34 (0.53)	10.11 (0.6)	10.41 (0.5)	10.65 (0.59)	0.033
CXCL9	7.51 (1.01)	6.98 (0.69)	7.51 (1.04)	8.05 (1.2)	0.043
PlGF	6.63 (0.37)	6.61 (0.51)	6.75 (0.44)	6.87 (0.51)	0.059
TNFRSF4	3.84 (0.32)	3.73 (0.51)	3.9 (0.69)	4.15 (0.72)	0.06
CDKN1A	0.03 (0.43)	0.1 (0.43)	0.27 (0.7)	0.3 (0.49)	0.067
NEMO	5.31 (0.72)	5.53 (0.77)	5.53 (0.63)	5.73 (0.7)	0.068
EMMPRIN	7.3 (0.22)	7.35 (0.22)	7.24 (0.21)	7.19 (0.37)	0.087
MIC.A	3.97 (1.78)	4.04 (1.42)	4.15 (1.21)	4.74 (1.3)	0.091
SELE	6.46 (0.72)	6.32 (0.79)	6.2 (0.56)	6.12 (0.83)	0.109
PRL	4.83 (1.16)	5.21 (0.89)	5.53 (0.92)	5.26 (1.2)	0.124
MIA	4.45 (0.43)	4.35 (0.32)	4.54 (0.41)	4.17 (0.52)	0.134
Ep.CAM	11.42 (0.96)	10.13 (0.91)	10.63 (1.1)	10.8 (0.78)	0.147
VEGF.D	7.01 (0.4)	7 (0.36)	7.01 (0.32)	6.85 (0.32)	0.162
IL.12	8.93 (0.49)	8.93 (0.48)	8.99 (0.63)	9.2 (0.88)	0.176
THPO	3.58 (0.42)	3.65 (0.34)	3.78 (0.57)	3.7 (0.37)	0.231
IL.6RA	6 (0.35)	6.11 (0.38)	6.04 (0.44)	5.85 (0.54)	0.243
ErbB3.HER3	9 (0.32)	9.03 (0.19)	8.98 (0.23)	8.9 (0.36)	0.253
MYD88	1.19 (0.7)	1.26 (0.54)	1.3 (0.61)	1.39 (0.5)	0.263
TNF	-0.62 (0.53)	-0.67 (0.4)	-0.64 (0.37)	-0.77 (0.06)	0.265
REG.4	3.58 (0.4)	3.26 (0.39)	3.45 (0.45)	3.38 (0.35)	0.266
GH	8.25 (1.51)	9.4 (1.55)	8.89 (1.71)	8.94 (1.09)	0.272
FAS	8.05 (0.33)	7.84 (0.39)	8.02 (0.43)	8.17 (0.68)	0.287
CXCL5	10.63 (0.88)	10.6 (0.83)	10.61 (0.81)	10.88 (0.52)	0.322
PECAM.1	5.92 (0.32)	6.02 (0.44)	5.9 (0.26)	5.84 (0.46)	0.34
IFN.gamma	0.89 (0.76)	0.82 (0.74)	1.34 (1.1)	0.98 (0.59)	0.351
CAIX	2.6 (0.76)	2.39 (1.16)	2.92 (1.13)	2.76 (1.22)	0.361
VE.statin	-0.06 (0.37)	0.23 (0.62)	0.19 (0.42)	0.06 (0.4)	0.432
EPO	-0.01 (1.17)	-0.02 (0.79)	-0.22 (0.53)	-0.15 (0.49)	0.444
TR.AP	5.32 (0.37)	5.19 (0.42)	5.3 (0.29)	5.37 (0.39)	0.51
Flt3L	8.4 (0.45)	8.13 (0.59)	8.41 (0.55)	8.43 (0.54)	0.546
CEA	1.95 (0.93)	1.92 (0.92)	2.09 (0.69)	1.74 (1.14)	0.611
TIE2	5.52 (0.35)	5.55 (0.28)	5.5 (0.22)	5.49 (0.43)	0.679
eIF.4B	2.86 (1.04)	2.73 (0.98)	2.87 (1.31)	2.95 (1.14)	0.71
ErbB2.HER2	9.2 (0.32)	9.11 (0.25)	9.23 (0.35)	9.13 (0.51)	0.823
IL.2	0.02 (0.2)	0.08 (0.52)	0.16 (0.51)	-0.03 (0.12)	0.831
PARK7	7.1 (0.48)	6.73 (0.37)	6.94 (0.48)	7.06 (0.48)	0.894
