# Supplementary material for: A multiplex platform for the identification of ovarian cancer biomarkers
Source: Clin Proteomics. 2017 Oct 10;14:34. doi: 10.1186/s12014-017-9169-6 (PMC5634875; doi:10.1186/s12014-017-9169-6)
Supplement: Supplementary file 3 — Additional file 3. AUC and sensitivity for all 92 proteins comparing late stage ovarian cancer versus benign. Comparison of Proseek® Oncology I values for serum samples from late stage high grade serous ovarian cancer patients versus women with benign ovarian conditions. [file 12014_2017_9169_MOESM3_ESM.pdf]

Additional file 3.

Protein	AUC (95% CI)	Rank	Sensitivity at 95% Specificity (95% CI)	Rank
HE4	1 (0.98, 1)	1	0.99 (0.91, 1)	2
CA.125	1 (0.97, 1)	2	1 (0.89, 1)	1
KLK6	0.95 (0.91, 0.99)	3	0.88 (0.74, 0.97)	3
MK	0.95 (0.9, 0.99)	4	0.79 (0.61, 0.96)	4
hK11	0.93 (0.87, 0.97)	5	0.7 (0.48, 0.91)	6
FR.alpha	0.91 (0.83, 0.98)	6	0.75 (0.55, 0.93)	5
IL.6	0.91 (0.82, 0.97)	7	0.69 (0.44, 0.88)	7
CSF.1	0.87 (0.75, 0.96)	8	0.4 (0.12, 0.8)	20
CXCL13	0.86 (0.77, 0.95)	9	0.6 (0.32, 0.82)	9
EZR	0.86 (0.76, 0.93)	10	0.63 (0.42, 0.83)	8
PRSS8	0.86 (0.74, 0.94)	11	0.56 (0.32, 0.81)	12
FUR	0.84 (0.74, 0.94)	12	0.48 (0.27, 0.76)	15
AM	0.83 (0.71, 0.94)	13	0.46 (0.22, 0.77)	17
CXCL10	0.83 (0.73, 0.92)	14	0.57 (0.32, 0.8)	10
TNF.R1	0.8 (0.67, 0.93)	15	0.36 (0.09, 0.75)	23
CSTB	0.79 (0.64, 0.92)	16	0.36 (0.11, 0.76)	22
ILT.3	0.79 (0.65, 0.91)	17	0.32 (0.09, 0.7)	26
CXCL9	0.78 (0.65, 0.9)	18	0.48 (0.21, 0.74)	16
VEGF.A	0.77 (0.65, 0.88)	19	0.52 (0.26, 0.71)	14
IL.7	0.76 (0.62, 0.89)	20	0.38 (0.14, 0.64)	21
ITGA1	0.76 (0.62, 0.88)	21	0.57 (0.38, 0.75)	11
ErbB4.HER4	0.75 (0.62, 0.86)	22	0.53 (0.32, 0.7)	13
IL.8	0.74 (0.56, 0.9)	23	0.2 (0.01, 0.66)	50
MCP.1	0.74 (0.59, 0.89)	24	0.22 (0.06, 0.57)	43
U.PAR	0.74 (0.57, 0.85)	25	0.15 (0.03, 0.41)	58
LYN	0.73 (0.58, 0.89)	26	0.3 (0.1, 0.57)	29
TNF.R2	0.73 (0.57, 0.87)	27	0.28 (0.06, 0.62)	33
CDH3	0.73 (0.57, 0.86)	28	0.36 (0.17, 0.6)	24
NTRK3	0.72 (0.58, 0.84)	29	0.42 (0.2, 0.64)	18
TRAIL.R2	0.72 (0.57, 0.87)	30	0.21 (0.02, 0.62)	45
Ep.CAM	0.71 (0.54, 0.87)	31	0.14 (0.01, 0.5)	59
FS	0.71 (0.55, 0.91)	32	0.17 (0.01, 0.64)	52
PARK7	0.71 (0.56, 0.84)	33	0.28 (0.1, 0.56)	32
SCF	0.7 (0.52, 0.88)	34	0.25 (0.01, 0.71)	39
FADD	0.7 (0.54, 0.82)	35	0.35 (0.14, 0.56)	25
CXCL11	0.69 (0.54, 0.87)	36	0.14 (0, 0.66)	60
EGFR	0.69 (0.53, 0.82)	37	0.28 (0.09, 0.53)	35
PDGF.subunit.B	0.69 (0.53, 0.85)	38	0.15 (0.03, 0.46)	56
VIM	0.69 (0.53, 0.82)	39	0.05 (0.01, 0.23)	84
ICOSLG	0.69 (0.55, 0.85)	40	0.41 (0.22, 0.62)	19
TNFSF14	0.69 (0.53, 0.86)	41	0.27 (0.12, 0.5)	37
MMP.1	0.69 (0.55, 0.84)	42	0.2 (0.06, 0.42)	47
TNFRSF4	0.68 (0.53, 0.82)	43	0.29 (0.04, 0.57)	30
GDF.15	0.68 (0.51, 0.88)	44	0.05 (0, 0.56)	85
BAFF	0.68 (0.5, 0.91)	45	0.11 (0, 0.67)	67

HGF	0.67 (0.51, 0.84)	46	0.2 (0.02, 0.63)	48
IL.1ra	0.66 (0.5, 0.84)	47	0.17 (0.03, 0.45)	53
FAS	0.66 (0.49, 0.81)	48	0.32 (0.05, 0.63)	27
FasL	0.66 (0.51, 0.81)	49	0.12 (0.01, 0.37)	63
HB.EGF	0.66 (0.5, 0.83)	50	0.16 (0.03, 0.46)	54
IL.6RA	0.65 (0.47, 0.8)	51	0.24 (0.07, 0.53)	41
CD69	0.65 (0.47, 0.8)	52	0.23 (0.06, 0.45)	42
Flt3L	0.65 (0.47, 0.81)	53	0.11 (0.02, 0.34)	70
EMMPRIN	0.64 (0.47, 0.79)	54	0.29 (0.1, 0.49)	31
MIC.A	0.64 (0.49, 0.82)	55	0.1 (0, 0.33)	71
PlGF	0.64 (0.47, 0.81)	56	0.13 (0, 0.54)	62
CD40.L	0.64 (0.45, 0.85)	57	0.28 (0.14, 0.53)	34
LITAF	0.64 (0.48, 0.79)	58	0.13 (0.02, 0.38)	61
CASP.3	0.64 (0.48, 0.77)	59	0.26 (0.07, 0.48)	38
VEGFR.2	0.64 (0.49, 0.82)	60	0.08 (0.01, 0.41)	79
PTPN22	0.63 (0.46, 0.79)	61	0.09 (0.01, 0.28)	77
ErbB3.HER3	0.62 (0.45, 0.77)	62	0.31 (0.1, 0.56)	28
VEGF.D	0.62 (0.44, 0.79)	63	0.09 (0, 0.33)	76
CCL19	0.62 (0.45, 0.8)	64	0.11 (0.01, 0.56)	69
CDKN1A	0.62 (0.45, 0.79)	65	0.15 (0.02, 0.45)	57
TR.AP	0.62 (0.44, 0.8)	66	0.09 (0, 0.38)	74
PECAM.1	0.61 (0.44, 0.78)	67	0.12 (0.02, 0.31)	66
CXCL5	0.61 (0.43, 0.75)	68	0.02 (0, 0.14)	88
MIA	0.61 (0.46, 0.8)	69	0.25 (0.08, 0.48)	40
IL.12	0.61 (0.44, 0.77)	70	0.28 (0.11, 0.51)	36
TGF.alpha	0.6 (0.44, 0.78)	71	0.09 (0.01, 0.41)	75
IL.17RB	0.6 (0.44, 0.77)	72	0.16 (0.04, 0.42)	55
TNF	0.6 (0.41, 0.69)	73	0 (0, 0.09)	92
GH	0.6 (0.42, 0.79)	74	0.03 (0, 0.34)	87
REG.4	0.59 (0.42, 0.79)	75	0.07 (0, 0.31)	81
VE.statin	0.59 (0.42, 0.73)	76	0.02 (0, 0.19)	89
AR	0.59 (0.41, 0.73)	77	0.2 (0, 0.47)	49
CAIX	0.59 (0.42, 0.76)	78	0.1 (0, 0.52)	72
IL.2	0.59 (0.34, 0.68)	79	0 (0, 0.11)	91
NEMO	0.58 (0.41, 0.73)	80	0.06 (0.01, 0.2)	82
MYD88	0.57 (0.4, 0.75)	81	0.06 (0, 0.35)	83
SELE	0.57 (0.39, 0.77)	82	0.09 (0.01, 0.33)	73
IFN.gamma	0.57 (0.42, 0.75)	83	0.04 (0, 0.35)	86
eIF.4B	0.56 (0.38, 0.72)	84	0.11 (0.02, 0.29)	68
EPO	0.55 (0.36, 0.72)	85	0.01 (0, 0.07)	90
LAP.TGF.beta.1	0.55 (0.39, 0.74)	86	0.08 (0.01, 0.3)	80
TF	0.55 (0.37, 0.72)	87	0.21 (0.04, 0.44)	46
CEA	0.55 (0.38, 0.74)	88	0.12 (0.03, 0.27)	64
TIE2	0.55 (0.37, 0.7)	89	0.18 (0.06, 0.34)	51
THPO	0.54 (0.39, 0.73)	90	0.08 (0.01, 0.28)	78
ErbB2.HER2	0.52 (0.34, 0.69)	91	0.22 (0.03, 0.5)	44
PRL	0.51 (0.34, 0.66)	92	0.12 (0.02, 0.35)	65
